# The Cardinal protocol: investigating a simplified, cost-conscious option for oocyte cryopreservation in a prospective noninferiority cohort study

**DOI:** 10.1016/j.xfre.2026.05.001

**Published:** 2026-05-08

**Authors:** Esther H. Chung, Natasha Raj-Derouin, Jiaqi Zhang, Victoria Harbour, Metabel Markwei, Arian Khorshid, Brindha Bavan, Lusine Aghajanova, Ruth B. Lathi

**Affiliations:** a Division of Reproductive Endocrinology and Infertility, Department of Obstetrics and Gynecology, Stanford University School of Medicine, Stanford, California; b Department of Obstetrics and Gynecology, Kaiser Permanente, Los Angeles, California; c Department of Obstetrics and Gynecology, Stanford University School of Medicine, Stanford, California; d Department of Obstetrics and Gynecology, Stanford University School of Medicine, Stanford, California

**Keywords:** Low-cost, oocyte cryopreservation, progestin protocol, access-to-care, Cardinal protocol

## Abstract

**Objective:**

To determine if the novel cost-conscious and simplified Cardinal protocol could achieve noninferior clinical outcomes in planned oocyte cryopreservation (OC) cycles, compared with routine protocols.

**Design:**

Prospective noninferiority cohort study, nonrandomized

**Subjects:**

Patients undergoing planned OC were prospectively recruited at a single academic institution between July 2023 and August 2024.

**Exposure:**

Participants self-selected between the routine protocols vs. the Cardinal protocol. All participants paid for their cycles out-of-pocket or using insurance. The Cardinal protocol is limited to 2 ultrasounds (baseline and stimulation day [SD] 9, no bloodwork, standardized follicle-stimulating hormone (FSH) dosing (antimüllerian hormone ≤ 4: 300 IU, antimüllerian hormone > 4: 225 IU), scheduled progestin start on SD6 in place of an antagonist, and autotrigger when ≥3 follicles are projected to be ≥18 mm. Routine protocols (antagonist, microdose flare) include up to maximum-dose gonadotropins, 5–6 ultrasounds, and 3–5 blood draws.

**Main Outcome Measures:**

The primary outcome was the number of mature metaphase II oocytes (MIIs) retrieved. Secondary outcomes included the total number of oocytes retrieved and cryopreserved, premature ovulation, ovarian hyperstimulation syndrome (OHSS), cycle cancellation, and postretrieval surgical complications. Total charges including the cost of medications were noted. A postcycle survey compared satisfaction and sociodemographic factors influencing patient choice between the Cardinal vs. routine protocols.

**Results:**

Of 118 total participants, 60 enrolled in Cardinal and 58 opted for routine. No significant differences (i.e., in age, body mass index, race/ethnicity) existed between groups. A total of 137 cycles (70 Cardinal and 67 routine) were compared. The mean difference in MIIs between groups was +1.08 (95% CI −1.1 to 3.27), demonstrating noninferiority of the Cardinal protocol. Premature ovulation did not occur in either group. The OHSS rates were lower in Cardinal (2.9% vs. 23%); no other significant differences in adverse events were noted. The median charge for a Cardinal cycle was $5,004 lower than that of routine protocols. Postcycle surveys (73% response rate) demonstrated higher satisfaction with Cardinal at 85% vs. 65%, driven by cost and convenience.

**Conclusions:**

The Cardinal protocol appears to be a noninferior option to routine protocols. It considerably reduced charges by $5,000 without a decrease in MIIs retrieved, making it a cost-conscious protocol that should be seriously considered for OC cycles. The Cardinal protocol was overall satisfactory for patients who selected this over the routine protocols, notably across diverse sociodemographic groups.

**Trial registration:**

NCT05842070; https://clinicaltrials.gov/study/NCT05842070.

The 2020–2025 strategic plan of the American Society for Reproduction Medicine (ASRM) prioritized equitable, affordable access to reproductive healthcare, calling for cost-effective treatments (1, 2, 3). The 2018 ASRM Access to Care Summit’s White Paper stated specifically: “Our field needs to identify cost-effective treatments, even if their success rate is lower, that patients shut out by finances can afford (2).” Despite increased attention in our field to broaden access to care, methods to achieve affordability without significantly sacrificing efficacy have not been rigorously studied. Existing published data on the overall comparative effectiveness of low-cost in vitro fertilization (IVF) protocols remain sparse (4). One study in 2017 evaluated a university-based low-cost IVF program that used oral ovulation induction agents along with low-dose gonadotropins, limited ultrasound monitoring, and no laboratory tests, to provide treatment to 65 patients from low-resource immigrant communities (5). They achieved a 29% cumulative live birth rate per retrieval with a high cancellation rate of 33.5%. Its major limitation was its small sample size, with no further follow-up studies that have emerged since its initial publication.

Although there have been some efforts to broaden access to infertility treatment with IVF, there have been very limited efforts to expand access for patients desiring planned oocyte cryopreservation (OC) as a strategy for deferred reproduction. However, access-to-care to OC is increasingly relevant, especially given the increasing public awareness and demand, as seen by the 1,752% increase in OC cycles in the United States between 2010 and 2019 (6). With planned OC frequently not covered by insurance and excluded by state mandates, this can lead to greater socioeconomic disparities with regard to future reproductive options and outcomes. Outstandingly, current cost estimates for 1 OC cycle in the United States, including medications, range from $12,000–$24,000 based on the literature and institutional pricing (2, 7).

In response to this, considering the existing literature on cost-saving measures in IVF (i.e., the use of progestin in place of gonadotropin-releasing hormone [GnRH] antagonist) (4, 8), we designed the novel Cardinal protocol—a cost-conscious, simplified version of routine OC protocols. Its name references both its “Cardinal” approach and our institution’s official color. Its simplification derives from standardized gonadotropin dosing, oral progestin for ovulation suppression, limited ultrasounds, and no bloodwork. We then evaluated whether the Cardinal protocol could achieve noninferior clinical outcomes in OC cycles compared with our clinic’s routinely used autologous protocols. We hypothesized that the Cardinal protocol would offer noninferior clinical outcomes for patients with respect to the number of mature metaphase II oocytes (MIIs) retrieved, improve overall patient satisfaction, and decrease costs, as well as time required off work to complete treatment. Our findings could ultimately pave the way for broader adoption of cost-effective, standardized protocols, making fertility preservation more accessible to individuals who might otherwise be unable to afford it for financial or time-limiting reasons.

## Materials and methods

### Study design

This prospective noninferiority cohort study was conducted at Stanford Fertility and Reproductive Health and approved by the Stanford University Institutional Review Board (Protocol 69100). This study was registered under ClinicalTrials.gov with the identifier NCT05842070 (9). Patients were recruited from late July 2023 to August 2024, with a follow-up period of data collection on their OC cycles until January 2025.

### Study participants

Patients undergoing planned OC were prospectively recruited at a single academic institution between 2023 and 2024. Inclusion criteria were kept purposefully broad to enable access-to-care for our patient population. These included: ovary-bearing individuals aged 18–40 years, antimüllerian hormone (AMH) > 0.3 ng/mL and AMH < 7 ng/mL. Exclusion criteria included: AMH > 7 ng/mL or physician concern for risk of developing severe ovarian hyperstimulation syndrome (OHSS), severely diminished ovarian reserve defined as AMH < 0.3 ng/mL, a body mass index (BMI) > 45 kg/m^2^, contraindications to ovarian stimulation or outpatient oocyte retrieval under anesthesia, or recent diagnosis of malignancy or disease requiring immediate OC before starting gonadotoxic therapy.

All ovary-bearing individuals were counseled extensively, and informed electronic consents were obtained before participation. All participants voluntarily joined the study, and all outcomes were tracked until January 2025. Participants were allowed to choose between the routine protocols as prescribed by their primary physician vs. the Cardinal protocol, and this decision was communicated to their care team.

### The Cardinal protocol

The two protocols and their differences are outlined side-by-side in Table 1 and Supplemental Figure 1 (available online). Patients opted to be in the Cardinal protocol or in the routine antagonist or microdose flare protocols as selected by their physician.

**Table 1:** Differences between the Cardinal protocol and routine antagonist protocol.

Characteristic	Cardinal protocol^a^	Routine protocol – antagonist
No. of ultrasounds	2^a^ (Baseline + SD9)	Approximately 4–7 per provider discretion
Bloodwork	None^a^	Approximately 4–7 per provider discretion
FSH doses (including the FSH component of Menopur)	225 IU if AMH >4 ng/mL 300 IU if AMH <4 ng/mL	Up to 450 IU; dose adjustments during cycle per provider discretion
Prevent premature surge timing	Start standardized on SD6 (AM)	Individualized based on follicle size + estradiol
Medicine to prevent premature surge	Oral Provera 10 mg	Antagonist injections
Trigger timing	At least 3 follicles projected >18 mm (Autotrigger on SD9)	Provider discretion based on scans + bloodwork
Trigger choice/dose	5K hCG + 4 mg Lupron; Adjust hCG dose or Lupron-only trigger per provider discretion	5K hCG + 4 mg Lupron; Adjust hCG dose or Lupron-only trigger per provider discretion

a Provider can add scans/bloodwork when deemed medically necessary. FSH = follicle-stimulating hormone; hCG = human chorionic gonadotropin; SD9/6 = stimulation day 9/6.

The Cardinal protocol includes two ultrasounds (baseline and stimulation day [SD] 9), no bloodwork/laboratory tests, standardized follicle-stimulating hormone (FSH) dosing (AMH <4 ng/mL: 300 IU, AMH > 4 ng/mL: 225 IU), scheduled progestin start for ovulation suppression (Provera 10 mg starting morning of SD6), and autotrigger based on SD9’s monitoring scan (when >3 follicles are projected to be >18 mm). Clomiphene citrate (150 mg for 5 days) could be added from SD1–SD5 for patients with an AMH <1 ng/mL. A dual trigger of human chorionic gonadotropin (hCG, 5,000 IU) and a GnRH agonist (Lupron, 4 mg) was the standard at our clinic, but providers could modify the trigger dose and consider a Lupron-only trigger if there was concern for OHSS based on follicle number. If the Lupron-only trigger was used, providers could also add luteinizing hormone (LH) and progesterone (P4) serum level testing for the following day.

Although we recommended cost-conscious strategies for the Cardinal protocol, providers were allowed to add ultrasounds and/or bloodwork, when deemed medically necessary to make a treatment decision or for the patient’s safety. This included estradiol (E2) levels if there was a cyst noted on a baseline scan or if there was a high concern for moderate-to-severe OHSS on SD9. Additional scans were ordered if a patient had a levonorgestrel-releasing intrauterine device in place to help determine their stage in the menstrual cycle, as well as if the physician found it difficult to predict the best day for autotrigger if lead follicles on SD9 were <14 mm.

The routine clinic protocols were the antagonist protocol, which was most frequently used, and the microdose Lupron flare protocol. These routine protocols included up to max-dose (450 IU) gonadotropins and generally 5–7 ultrasounds and 3–5 blood draws. Gonadotropin dose changes were allowed per provider discretion. The antagonist injection was started flexibly, depending on follicle size > 14 mm or E2 > 500 pg/mL per provider discretion. Patients were triggered on the night of their last ultrasound, with triggers and doses decided by their physician. The dual trigger of hCG and Lupron was our clinic standard, and the Lupron-only trigger was recommended with an E2 level >6,000 pg/mL.

### Outcomes

The prespecified primary outcome was the mature oocyte yield per retrieval or the number of MIIs retrieved. Related secondary outcomes were the total number of oocytes retrieved, total number of MIIs and MIs-converted-to-MIIs (MI-MIIs) retrieved, and the total number of oocytes cryopreserved. Other clinically important secondary outcomes included premature ovulation, cycle cancellation, OHSS (including mild as defined by ASRM (10)), postretrieval surgical complications.

Total institutional billed charges (including the cost of medications) for each cycle were also noted. It is important to note that, excluding the cost of medications, these were charge data and not the final out-of-pocket costs the patient was responsible for. To estimate per-cycle charges, we calculated the total charges for each participant by multiplying the number of ultrasounds, laboratory draws, and medication fills by their respective unit charges (Supplemental Table 1, available online). These calculations were intended to approximate the full scope of cycle-related charges, including both monitoring and medication costs. We used our internal itemized fee structure to outline the per-unit charges for each of the cycle components, and specific line-item charges for the most pertinent clinic services and medications are presented. The relative difference in overall charges is provided below.

Postcycle survey compared satisfaction and sociodemographic factors influencing patient choice between the Cardinal vs. routine protocols. This also included self-reported out-of-pocket costs and hours spent away from work to try to better capture the patients’ real-world experience.

### Postcycle survey

The survey was disseminated through the research electronic data capture tools (REDCap) hosted at Stanford University (11). After retrieval, participants were emailed a 10-minute postcycle survey that compared satisfaction and sociodemographic factors that could potentially influence participant choice between the Cardinal and routine protocols. The survey collected general demographic information from participants, overall satisfaction with their protocol of choice, out-of-pocket costs, distance traveled, and time needed off work. Satisfaction was gauged through a Likert scale. A $50 gift card was provided for survey completion for their time and feedback.

### Statistical analyses

We tested the following hypothesis:

Null hypothesis (H_0_): μ(Cardinal)−μ(routine)≤−5 MIIs per retrieval

Alternative hypothesis (H_1_): μ(Cardinal)−μ(routine)>−5 MIIs per retrieval

The noninferiority margin of −5 MIIs per retrieval was selected based on existing literature on planned OC, which reports expected yields of 9–13 MIIs with substantial (approximately 45%) variability in ovarian stimulation response (12, 13, 14, 15). An absolute margin of −5 ensured that observed differences in oocyte yield would be beyond expected biologic variation, while still maintaining clinical viability. This noninferiority margin was reviewed and approved with consensus from all 15 physicians at our site. Prior noninferiority studies have used margins of 2–3; however, given the well-described approximately 45% intercycle and interindividual variability in oocyte yield even with identical stimulation protocols, such small margins would fall within expected biologic variation. We therefore selected −5 MIIs to ensure that our observations reflect a true protocol effect rather than normal variability.

The sample size was determined a priori. Using a one-sided test for noninferiority and assuming the standard deviation of 7.07 for the primary outcome of number of MIIs retrieved (11, 13), we calculated that a total of 52 participants (26 per group) was needed to achieve 80% power to detect a mean difference of −5 in number of MIIs. Although the sample size calculation indicated that 52 participants would be sufficient to detect noninferiority, we elected to set an enrollment target of 100 participants. This decision was driven by a pragmatic and ethical consideration: the Cardinal protocol was intentionally designed to reduce cost and increase access to OC. Given that the protocol posed no anticipated medical risks relative to standard protocols, we felt it was appropriate and ethical to expand enrollment and allow a larger number of interested patients to participate.

Given that some participants underwent multiple cycles, patient characteristics were summarized at the patient level. Five patients switched cycle protocol for their second oocyte retrieval. These patients’ groups were determined by their first cycle. The cycle characteristics and costs were presented at the cycle level. The postcycle survey results were summarized at the patient level. The differences between groups were compared by the Wilcoxon’s rank sum test for continuous variables, and the Pearson χ^2^ test or Fisher’s exact test for categorical variables, as appropriate.

Noninferiority was assessed by first calculating the mean and 95% confidence interval (CI) for the difference between the treatment groups for the primary outcome (number of MIIs retrieved) and the secondary continuous outcomes (total number of oocytes retrieved, total number of MIIs and MI-MIIs retrieved, and total number of oocytes cryopreserved). We then compared the lower bound of the CI with the predefined noninferiority margin of −5. We conducted these analyses using linear regression models fit with generalized estimating equations (geepack package in R). These models accounted for the within-subject correlation introduced by participants who underwent multiple cycles. The adjusted models included age, race (Asian, White, other, or unknown), ethnicity (Hispanic, non-Hispanic, unknown), BMI, antral follicle count (AFC), and AMH as covariates (16). Missing data were handled by including missing indicator variables in the models. A sensitivity analysis including only the first cycle for each participant was also performed using the above methods. Another subgroup analysis was performed using only normal responders with an AMH of 1–4. We further assessed differences between the treatment groups in secondary categorical outcomes (premature ovulation, cycle cancellation rate, OHSS, use of cabergoline, and postretrieval surgical complications) at the cycle level using the Pearson χ^2^ test or Fisher’s exact test as appropriate. Statistical analyses were performed using R 4.4.2.

## Results

A total of 331 people were introduced to the study from late July 2023 to August 2024 (Supplemental Fig. 2, available online); 26 were deemed ineligible to partake. Out of the 168 eligible participants who signed the consent form, 118 participants completed at least one cycle by January 2025. Of these, 60 chose the Cardinal protocol and 58 opted for a routine protocol. Seventeen participants underwent a second cycle, of whom two switched protocols from Cardinal to routine and three switched protocols from routine to Cardinal.

### Baseline characteristics

The participants’ baseline characteristics are presented in Table 2. No significant differences existed between groups. Median age was 33 years in Cardinal and 32 in routine (*P* =.067). Median AMH was 2.56 in Cardinal vs. 3.32 in routine (*P* =.055), and median AFC was 15 in Cardinal vs. 17 in routine (*P* =.093). The routine group had slightly higher median AMH and AFC values than the Cardinal, although these differences did not reach statistical significance. This may suggest that the routine group may have had a modestly more favorable baseline prognosis for their OC cycle. There were no differences in race/ethnicity (*P* =.91, *P* >.99 respectively), with high Asian representation across both (approximately 40%–45%). Median distance traveled to the clinic was similar across both groups (*P* =.65), although the maximum distance was notably further in the Cardinal. The Cardinal protocol included two patients from outside California and two patients residing outside the Bay Area, who specifically desired to do their cycles at our clinic and hence traveled for their cycle, whereas all patients in the routine protocol were located within the Bay Area.

**Table 2:** Patient baseline and cycle characteristics.

Characteristic	Cardinal	Routine	*P* value
Participants	60	58	–
Age	33 (32, 35)	32 (31, 34)	.07
AFC	15 (11, 19)	17 (12, 23)	.09
AMH (ng/mL)	2.56 (1.54, 3.55)	3.30 (1.86, 4.68)	.06
Age at retrieval			.07
Median (IQR)	33 (32, 35)	32 (31, 34)	
Min, max	21–39	27–40	
Race			
American Indian or Alaska native	0 (0%)	0 (0%)	.28
Asian	25 (45.5%)	26 (45.6%)	
Black or African American	3 (5.5%)	4 (7.0%)	
Native Hawaiian or other Pacific islander	0 (0%)	0 (0%)	
White	23 (41.8%)	19 (33.3%)	
Other	2 (3.6%)	7 (12.3%)	
More than one races	2 (3.6%)	1 (1.8%)	
Unknown	5	1	
Hispanic ethnicity			>.99
Yes	3 (5.3%)	3 (5.3%)	
No	54 (94.7%)	54 (94.7%)	
Unknown	3	1	
Distance from home to clinic (miles)			.65
Median (IQR)	14 (10, 38)	15 (7, 26)	
Min, max	2.4, 2938	2, 64.3	
Unknown	0	2	
Cycles	**70**	**67**	–
BMI	23.7 (21.6, 25.9)	23.3 (21.7, 26.3)	.72
Excluding 1 cancelled cycle	**70**	**66**	
Starting FSH dose (IU)	300 (300, 300)	375 (300, 450)	<.01
Daily LH dose (IU)	75 (75, 75)	150 (75, 150)	<.01
Total FSH dose (IU)	2,400 (2,400, 3,000)	3,075 (2,525, 4,106)	<.01
No. of US visits	2 (2,3)	5 (5, 6)	<.01
No. of total laboratory tests drawn	0 (0,2)	5 (4, 8)	<.01
Trigger day	10 (9,11)	10 (9,11)	.34
Total charges (including medications)	$19,774 (19,373, 20,543)	$24,778 (23,812, 26,075)	<.01

*Note:* Continuous variables were reported by median (25% quantile, 75% quantile) unless otherwise specified. AFC = antral follicle count; AMH = antimüllerian hormone; BMI = body mass index; FSH = follicle-stimulating hormone; IQR = interquartile range; LH = luteinizing hormone; US = ultrasound.

### Cycle characteristics

A total of 137 cycles (70 Cardinal and 67 routine) were initiated and reported for cycle characteristics. There was one cycle cancellation in the routine protocol group because of limited ovarian response. So, a total of 136 OC cycles were compared, with 70 Cardinal protocol cycles and 66 routine protocol cycles.

As per protocol, the median starting FSH and LH doses were significantly lower in the Cardinal group, than in the routine group (median [25% quantile, 75% quantile, interquartile range [IQR] 300 IU [300, 300] vs. 375 IU [300, 450]; *P* <.001 and 75 IU [75, 75] vs. 150 IU [75, 150]; *P* <.001, respectively). Total FSH dose used during the cycle was also significantly lower in the Cardinal group (2400 IU [2400, 3000] vs. 3075 IU [2525, 4106]; *P* <.001).

The Cardinal group required fewer ultrasound visits (median [IQR] 2 [2,3] vs. 5 [5,6]; *P* <.001), and fewer total laboratory tests (0 [0,2] vs. 5 [4,8]; *P* <.001), reflecting a significant reduction in monitoring burden.

There was, however, no significant difference in the median day of trigger between the 2 groups (median [IQR] SD 10 [9, 11] vs. SD 10 [9, 11]; *P* =.341).

### Cycle outcomes

Adjusted for age, BMI, race, ethnicity, AFC, and AMH, the mean difference in the number of MIIs retrieved between groups (Cardinal – Routine) was +1.08 (95% CI −1.1 to 3.27) (Table 3, Supplemental Fig. 3, available online). The lower bound of the CI did not cross the prespecified noninferiority margin of −5 MIIs, demonstrating noninferiority of the Cardinal protocol to the routine protocols. Noninferiority was demonstrated in all other oocyte number-relevant outcomes, with the mean difference of the number of all oocytes retrieved at +0.71 (95% CI: −1.94 to 3.4), the number of MIIs + MI-MIIs at +1.03 (95% CI: −1.26 to 3.31), and the number of all oocytes cryopreserved at +1 (95% CI: −1.21 to 3.22).

**Table 3:** Cardinal vs. routine cycle: number of oocytes and secondary clinical outcomes.

Outcome	Cardinal (No. of cycles = 70)	Routine (No. of cycles = 66)	Crude mean difference (95% CI)	Adjusted mean difference^a^ (95% CI)
No. of oocytes retrieved	13 (8, 20)	16 (11, 24)	−1.6 (−4.6, 1.4)	0.71 (−1.94, 3.4)
No. of MIIs retrieved	10 (7, 15)	11 (7, 16)	−0.64 (−3.2, 1.9)	1.08 (−1.1, 3.27)
No. of MIIs and MI–MII	10 (7, 15)	12 (8, 17)	−0.68 (−3.3, 1.9)	1.03 (−1.26, 3.31)
No. of oocytes frozen	10 (7, 16)	12 (8, 17)	−0.77 (−3.3, 1.8)	1 (−1.21, 3.22)

Outcome	Cardinal (No. of cycles = 70)	Routine (No. of cycles = 67)	*P* value
Cycle cancellation	0	1 (1.5%)	0.49

Outcome	Cardinal (No. of cycles = 70)	Routine (No. of cycles = 66)	*P* value
Premature ovulation	0 (0%)	0 (0%)	/
OHSS (includes mild)	2 (2.9%)	15 (22.7%)	<.01
Moderate or severe OHSS	1 (1.4%)	4 (6.1%)	.52
Ovarian torsion	0 (0%)	0 (0%)	/
Postop clinic visit	1 (1.5%)	4 (6.1%)	.20
Postop ED visit	0 (0%)	2 (3.0%)	.23
Surgical complications	0 (0%)	0 (0%)	/

a Adjusted for age, BMI, race, ethnicity, AFC and AMH. AFC = antral follicle count; AMH = antimüllerian hormone; BMI = body mass index; CI = confidence interval; ED = emergency department; MI/MII = metaphase I/metaphase II; OHSS = ovarian hyperstimulation syndrome.

Regarding the categorical secondary clinical outcomes (Table 3), there were fewer occurrences of OHSS in the Cardinal group (2.9% vs. 23%, *P* <.001). However, no significant difference was noted when mild OHSS cases were excluded. Cabergoline was prescribed in 17% of the Cardinal group vs. 26% in the routine group (*P* =.22). There was one cycle cancellation in the routine group because of patient preference in the setting of limited ovarian response (0% vs. 1.5%, *P* =.489). Premature ovulation did not occur in either group. After retrieval, there were no identified cases of ovarian torsion or surgical complications (bleeding, infection, injury to surrounding structures) There were more postoperative clinic and emergency room visits in the routine group, but these were not statistically significantly different (1.5% vs. 6.1%; *P* =.199 and % vs. 3.0%; *P* =.234, respectively).

With respect to the five participants who switched cycle protocols for their second oocyte retrieval, four demonstrated outcomes with Cardinal that were either slightly better than or equivalent to those from the routine cycle. One participant had notably poorer outcomes with the Cardinal protocol compared with the routine, but results for this transgender male participant were potentially impacted by the duration of time from testosterone (T) cessation, where he had been 3–4 months off T when he opted for Cardinal vs. 6 months off T when he switched to routine.

Although repeat cycles were uncommon, given the potential for bias from including repeat cycles, we conducted a sensitivity analysis limited to the first cycles only. These analyses yielded comparable results to the full dataset. The adjusted mean difference in MIIs retrieved between groups (Cardinal – routine) was +0.64 (95% CI: −1.7 to 3.0) (Supplemental Tables 2–4, available online). In another secondary analysis restricted to those <35 years with AMH 2–4 ng/mL (good prognosis patients), the number of MII oocytes retrieved remained similar between the Cardinal and routine groups (adjusted difference Cardinal – routine was +2.1 (−2.7, 5.8)), consistent with our primary findings.

Another subgroup analysis was performed, limiting cycles for normal responders defined as an AMH of 1–4. The subgroup comprised 72 normal responders, corresponding to 80 cycles. In the normal responders, 42 selected Cardinal and 38 selected routine. The findings from this subgroup were consistent with the results obtained from the full cohort analysis and can be found in Supplemental Table 5 (available online). The Cardinal protocol demonstrated noninferiority to the routine protocol with respect to key outcomes, including the number of oocytes retrieved, the number of MIIs retrieved, the number of MI-MIIs and MIIs, and the number of oocytes frozen.

### Charge outcomes

**Figure 1:**
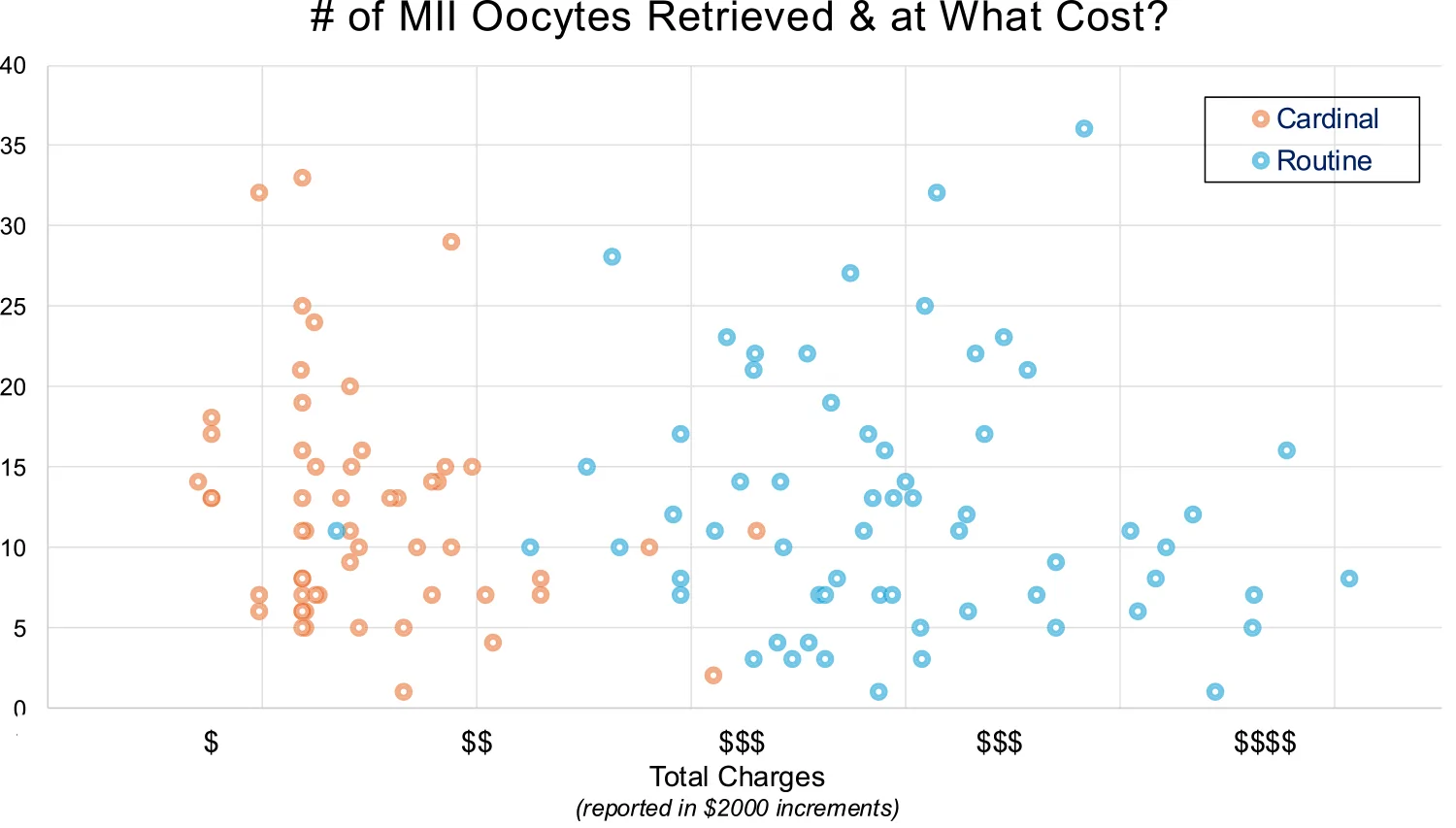
Number of metaphase II oocytes retrieved and at what cost.?

The median total charges for the Cardinal protocol cycles were $5,004 (*P* <.001) less than that of the routine protocols, which reflected an overall 20% decrease in charges. Figure 1 demonstrates that there is clear cost-savings without a decrease in MIIs retrieved. As expected, with an established cost-conscious protocol in place, the variability in total charges for cycles completed using the Cardinal protocol was much smaller compared with the routine protocols.

### Postcycle survey outcomes

Postcycle surveys were completed by 86 participants (46 from the Cardinal group and 40 from the routine), reaching a 72.9% response rate. Responses were similar across the 2 groups: 46/60 (76%) and 40/58 (69%). See Supplemental Table 6 (available online) and Supplemental Figure 4 (available online).

Overall satisfaction, defined as participants selecting 4 or 5 out of 5-point Likert scale, was significantly higher in the Cardinal group (85% vs. 65% in routine; *P* =.033). This difference was primarily attributed to cost and convenience factors. Dissatisfaction with cost and convenience was higher in the routine group (39% vs. 63%, *P* =.031, 13% vs. 48%, *P* <.001, respectively). No differences in patient attitudes toward ovarian response/outcome (number of oocytes) or toward staff were noted between groups.

Level of insurance coverage (complete, partial, or none) appeared similar between groups (*P* =.6). However, more Cardinal participants reported annual household incomes less than $200,000 (83% vs. 55%, *P* =.005). Current employment status was also assessed and found to be similar between the 2 groups (*P* =.62).

Excluding the day of retrieval, the median number of hours needed off work was twice as high in the routine group at 10 hours (IQR, 6–16) vs. 5 hours (IQR, 3–12) in the Cardinal group (*P* =.025).

Statistical testing did not reveal other significant differences between the groups. The following trends should be interpreted with caution, because the study was not powered to detect differences across categorical variables with multiple levels. Regarding sexual orientation and gender identity, several transmen specifically selected the Cardinal protocol to limit the number of transvaginal scans to reduce gender dysphoria and discomfort. Of the survey respondents, 20% of Cardinal participants identified as not heterosexual vs. 7.5% in the routine group (*P* =.11).

## Discussion

The Cardinal protocol appears to be a noninferior, cost-conscious option compared with routine protocols. Our noninferiority analyses show that not only did the lower bounds of the CI not cross the prespecified margin, but also that all adjusted point estimates for oocyte outcomes slightly favored the Cardinal group. This could suggest potential equivalence or even mild superiority. However, the study was neither designed nor powered to detect the latter, and these findings should be interpreted as hypothesis-generating.

The Cardinal protocol was designed to combine several cost-saving levers identified in the literature, embodying a simplified, cost-conscious IVF protocol. We review here the rationale behind the elements that comprise the Cardinal protocol. Regarding the use of a progestin in lieu of GnRH antagonist, multiple prospective studies and randomized controlled trials (RCTs) demonstrate Provera’s comparable efficacy at preventing ovulation with similar reproductive outcomes, its affordability, convenience, and reduced complexity (8, 17, 18, 19, 20, 21, 22, 23, 24). Not only is it an oral medication in place of an injectable, it also carries the potential to decrease the number of visits during stimulation, justifying its use in our protocol. Unfortunately, progestin use was not part of routine practice at our institution during the study period, so its impact on cost and convenience was outside the scope of this study.

One of the largest out-of-pocket cost drivers in IVF is medication (25). We decided to use a standardized AMH-based dosing of 225–300 IU/d of FSH instead of tailoring doses by AMH/AFC up to a maximum of 450 IU/d. A recent RCT demonstrated that individualized FSH dosing based on AFC did not improve live birth rates and adversely increased costs compared with a standardized 150 IU/d (26). A meta-analysis of 2,000 cycles similarly suggested that although higher daily FSH doses >200 IU/d may increase oocyte yield, they do not necessarily improve embryo availability or pregnancy outcomes in normal responders (27). Notably, the routine protocol group received higher daily and total FSH doses compared with the Cardinal group, which likely contributed to the increased medication-related costs observed in that group. Accordingly, some of the cost difference between protocols may reflect differences in stimulation intensity rather than protocol structure alone. Also, as medication pricing continues to evolve since the study period, this may potentially attenuate the absolute cost differences observed between protocols in the future.

Evidence on whether dual trigger (GnRH agonist + hCG) improves oocyte and embryo parameters or live birth rates remains mixed (28, 29, 30, 31). Given the added cost of approximately $175 per cycle, our clinic elected to routinely use the dual trigger when feasible, although we acknowledge that the supporting data are not definitive (32). Considering this minimal increase in cost relative to the overall high cost of IVF, the dual trigger, which was our preexisting clinic standard-of-care, was considered a cost-effective strategy to incorporate into the Cardinal protocol.

A major limitation of this study is its nonrandomized design. An RCT would have provided stronger evidence; however, financial constraints would have made that study design unfeasible. Additionally, participant and survey reporting bias may limit our interpretation of these results, but the Cardinal vs. routine population appeared similar across baseline characteristics. The trend toward higher AFC and AMH in the routine group could be a confounder, but this imbalance biases against the Cardinal protocol, actually strengthening the observed noninferiority findings. Patients with diminished ovarian reserve, who may have anticipated multiple OC cycles, may have been more likely to choose the lower-cost, lower-lift Cardinal protocol. Still, we must limit causal inference because of residual confounding, and, ultimately, our findings warrant validation in a randomized study.

Another potential limitation may lie in the difference in how gonadotropin dosing was determined across protocols. In the routine group, dosing was left to the discretion of individual physicians, who often favored higher doses to optimize oocyte yield; however, this approach may not be generalizable to other practice settings. This variation in physician-driven dosing may also have contributed to the higher observed incidence of OHSS in the routine protocol group. However, given the retrospective, nonrandomized design and the subjective nature of OHSS diagnosis in clinical practice, this finding should be interpreted with caution. Differences in clinical ascertainment, documentation, and observer variability may have influenced reported OHSS rates. Because E2 levels were not routinely measured in the Cardinal protocol, objective hormonal differences could not be assessed. Accordingly, the observed OHSS difference should be considered hypothesis-generating rather than evidence of a causal relationship between protocol type and OHSS risk.

Another major limitation is that our charge data may not portray the most accurate picture of the patient-facing financial experience because we were limited by a lack of visibility into each individual’s insurance coverage. However, we used uniformly billed institutional charges to calculate per-cycle costs based on the number of services rendered, rather than patient out-of-pocket expenses. This approach represented the most objective and consistent method available to compare costs between protocols, given limitations of data accessibility, but it contributes to the higher absolute charges observed even in the lower-cost protocol. Ideally, the most accurate measure would involve final billing data from the billing department or collecting itemized receipts reflecting actual payments made by patients to both the clinic and pharmacies. We recommend incorporating such data collection strategies in future studies focused on patient-incurred treatment costs.

Generalizability may be a concern because the study disproportionately represents individuals of Asian race compared with the US population (7.1%) (33). However, we do not believe these demographic differences significantly impact generalizability, as we observed no racial difference in protocol selection or outcomes. Black (5.9%) and Hispanic (5.1%) representation were consistent with prior Society for Assisted Reproductive Technology utilization analyses (33, 34, 35, 36). Another point on generalizability across all practice settings is that many clinics use bundled, all-inclusive pricing for each cycle, as opposed to a fee-for-service schedule. This may obscure protocol-specific monitoring cost differences. Even still, reduced monitoring under a simplified protocol may allow clinics to increase throughput and appointment availability. For the patient, fewer visits, laboratory tests, and medication requirements translate to lower downstream out-of-pocket expenses, especially in settings without global fees.

Ultimately, we believe that the study’s inclusive nature facilitated broader access to fertility preservation, particularly for patients with demanding schedules, limited insurance coverage, multiple planned cycles, or long travel distances. The reduced-monitoring burden may also lessen barriers for transmen patients who may experience gender dysphoria with frequent transvaginal ultrasounds (37), and reduce the injection burden for individuals with needle phobia (38).

So, can the Cardinal protocol be applied for embryo cryopreservation for fertility preservation? Or for freeze-all IVF cycles for patients using preimplantation genetic testing for aneuploidy? At our own clinic, our providers’ experience and familiarity with the use of the Cardinal protocol have led to a more seamless adoption of progestin protocols for freeze-all IVF cycles as a cost-conscious approach. Beyond reducing cycle and medication costs, adoption of this protocol may increase provider confidence in minimizing unnecessary bloodwork and extra ultrasounds, while still achieving comparable clinical outcomes. We acknowledge that aspects of our ‟routine” protocol reflect our institutional practice and may not represent ‟routine” care across all clinics, a variability inherent to our field. But this study’s primary contribution is demonstrating that a simplified, reduced-monitoring approach was associated with comparable clinical outcomes while lowering costs and resource utilization. Well-designed studies should continue to evaluate strategies that maintain similar clinical efficacy while reducing cost, optimizing the patient experience, and increasing provider experience with less intensively monitored IVF cycles.

## Conclusion

Based on this retrospective, nonrandomized cohort, the Cardinal protocol was associated with noninferior mature oocyte yields comparable with those of routine protocols; however, further validation through randomized trials is warranted to confirm this finding. Overall, it minimized the number of monitoring visits, ultrasounds, blood draws/laboratory tests, and injections to what was deemed medically necessary. It also considerably reduced total charges by approximately $5,000, making it a cost-conscious protocol for OC cycles. Not only did the Cardinal protocol decrease cost and cost variability per cycle, but it also improved patient satisfaction compared with the routine protocols. Offering simplified, cost effective, and patient-centered protocols like Cardinal can play a meaningful role in breaking down barriers to fertility care and expanding access to a broader patient population.

## CRediT Authorship Contribution Statement

**Esther H. Chung:** Writing – review & editing, Writing – original draft, Visualization, Project administration, Methodology, Investigation, Funding acquisition, Data curation, Conceptualization. **Natasha Raj-Derouin:** Writing – review & editing, Project administration, Investigation, Data curation. **Jiaqi Zhang:** Writing – review & editing, Software, Resources, Formal analysis, Data curation. **Victoria Harbour:** Writing – review & editing, Investigation, Data curation. **Metabel Markwei:** Writing – review & editing, Investigation, Data curation. **Arian Khorshid:** Writing – review & editing, Formal analysis. **Brindha Bavan:** Writing – review & editing, Methodology, Investigation. **Lusine Aghajanova:** Writing – review & editing, Methodology, Investigation. **Ruth B. Lathi:** Writing – review & editing, Supervision, Methodology, Investigation, Funding acquisition, Conceptualization.

## Declaration of Interests

E.H.C. is an advisor for LEVY Health (no exercised stock options). N.R.D. has nothing to disclose. J.Z. has nothing to disclose. V.H. reports Med Scholars Research Grant – Stanford University outside the submitted work. M.M. has nothing to disclose. A.K. has nothing to disclose. B.B. reports travel support from Stanford University OB/GYN Department. L.A. reports SREI Board and PCRS Board of Directors. R.B.L. reports this project was supported by internal REI division funding through Ruben Alvero MD.

## Supplementary Data

Supplementary Tables 1-6

Supp Fig 3_Cardinal v Routine

Supplemental Figure 4_SEFS_EC_Final
