# Supplementary material for: The Cardinal protocol: investigating a simplified, cost-conscious option for oocyte cryopreservation in a prospective noninferiority cohort study
Source: F S Rep. 2026 May 8;7(4):301–12. doi: 10.1016/j.xfre.2026.05.001 (PMC13494622; doi:10.1016/j.xfre.2026.05.001)

Supplemental Figure 3: Cardinal vs. Routine Non-Inferiority Assessment, Adjusted Mean Difference in Oocyte Yields

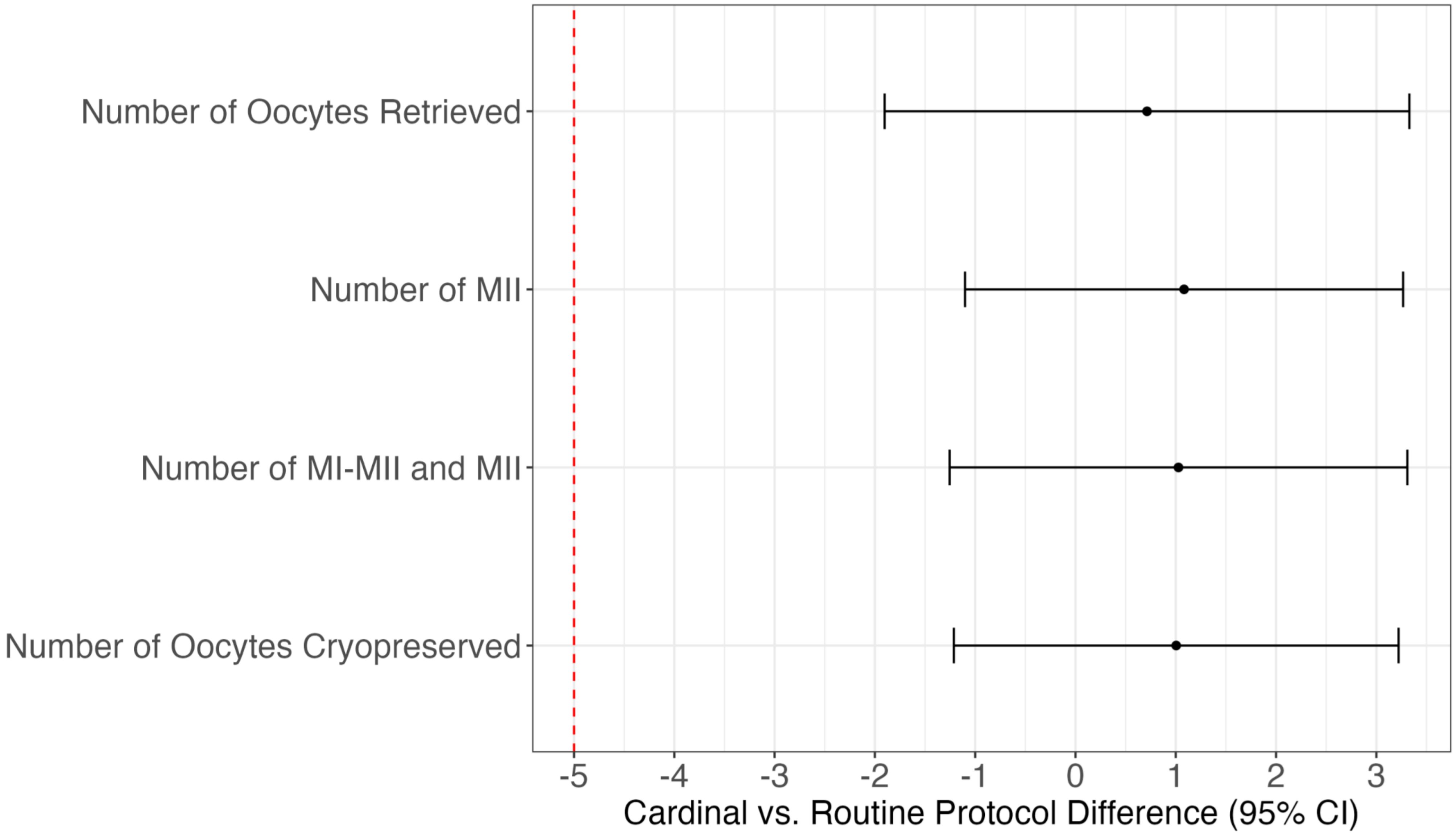

Supplement: Supplementary file 1 [file mmc2.pdf]
