# Supplementary material for: The Cardinal protocol: investigating a simplified, cost-conscious option for oocyte cryopreservation in a prospective noninferiority cohort study
Source: F S Rep. 2026 May 8;7(4):301–12. doi: 10.1016/j.xfre.2026.05.001 (PMC13494622; doi:10.1016/j.xfre.2026.05.001)
Supplement: Supplementary file 2 [file mmc3.pdf]

**Supplementary Figure 4:** Post-cycle Survey Outcomes on Patient Satisfaction and Socio-demographic Factors

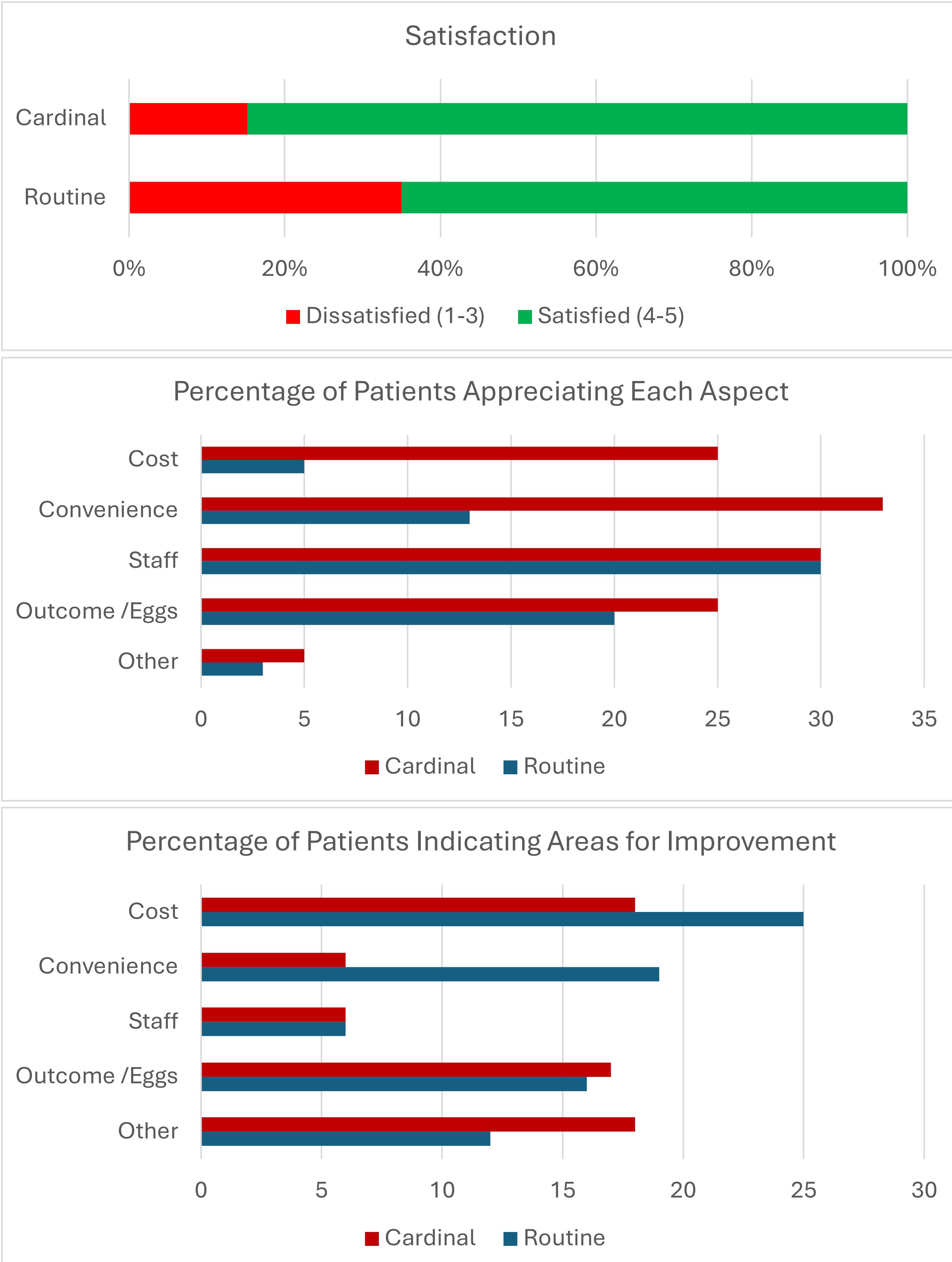

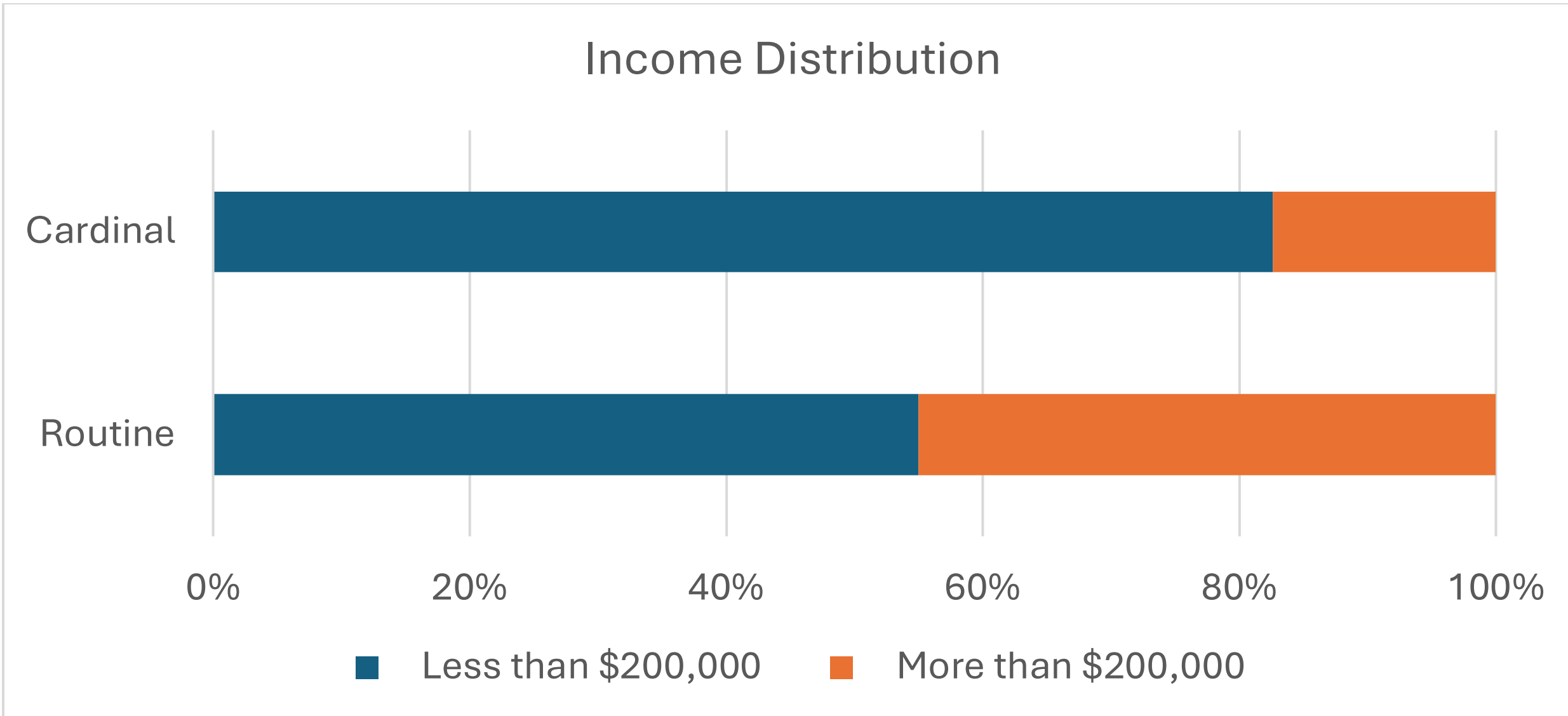
Income Distribution
Cardinal
Routine
0%
20%
40%
60%
80%
100%
Less than $200,000
More than $200,000

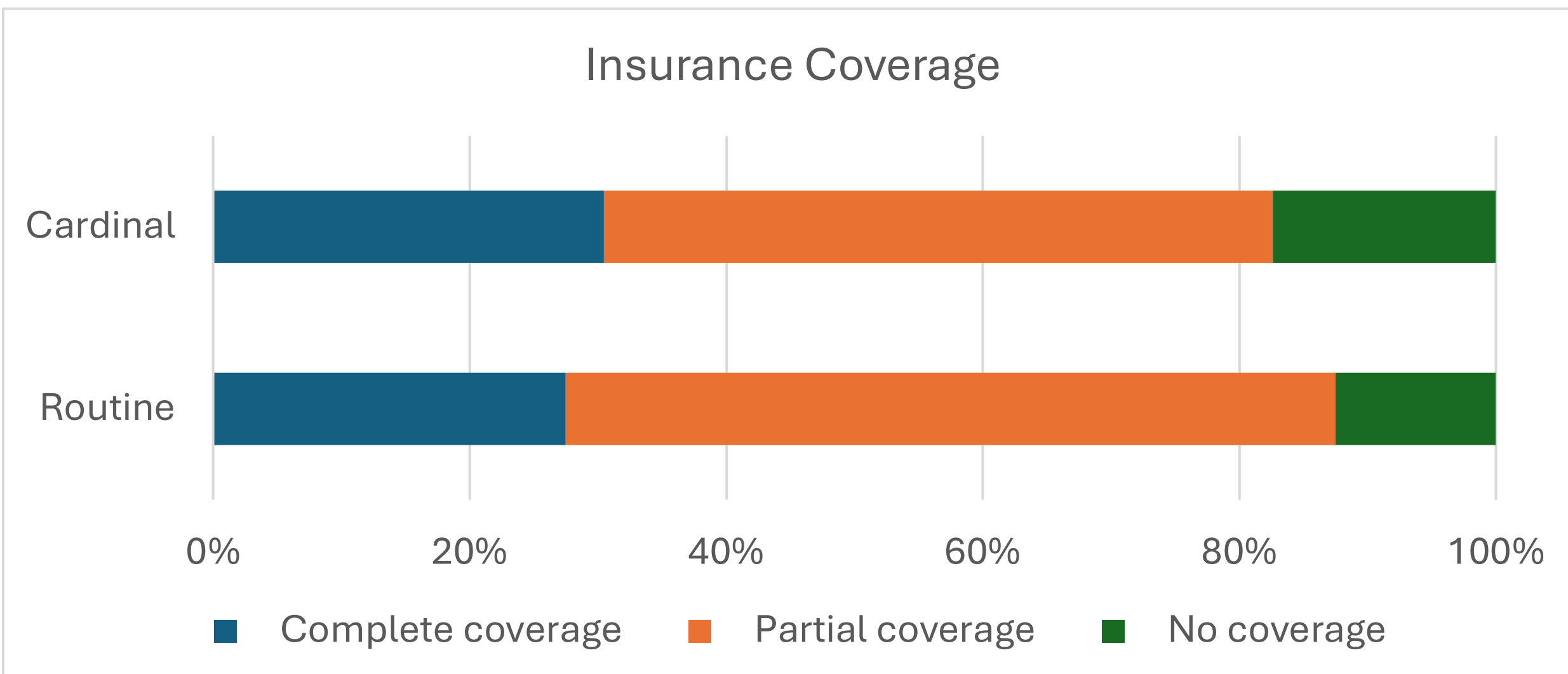
Insurance Coverage
Cardinal
Routine
0%
20%
40%
60%
80%
100%
Complete coverage
Partial coverage
No coverage

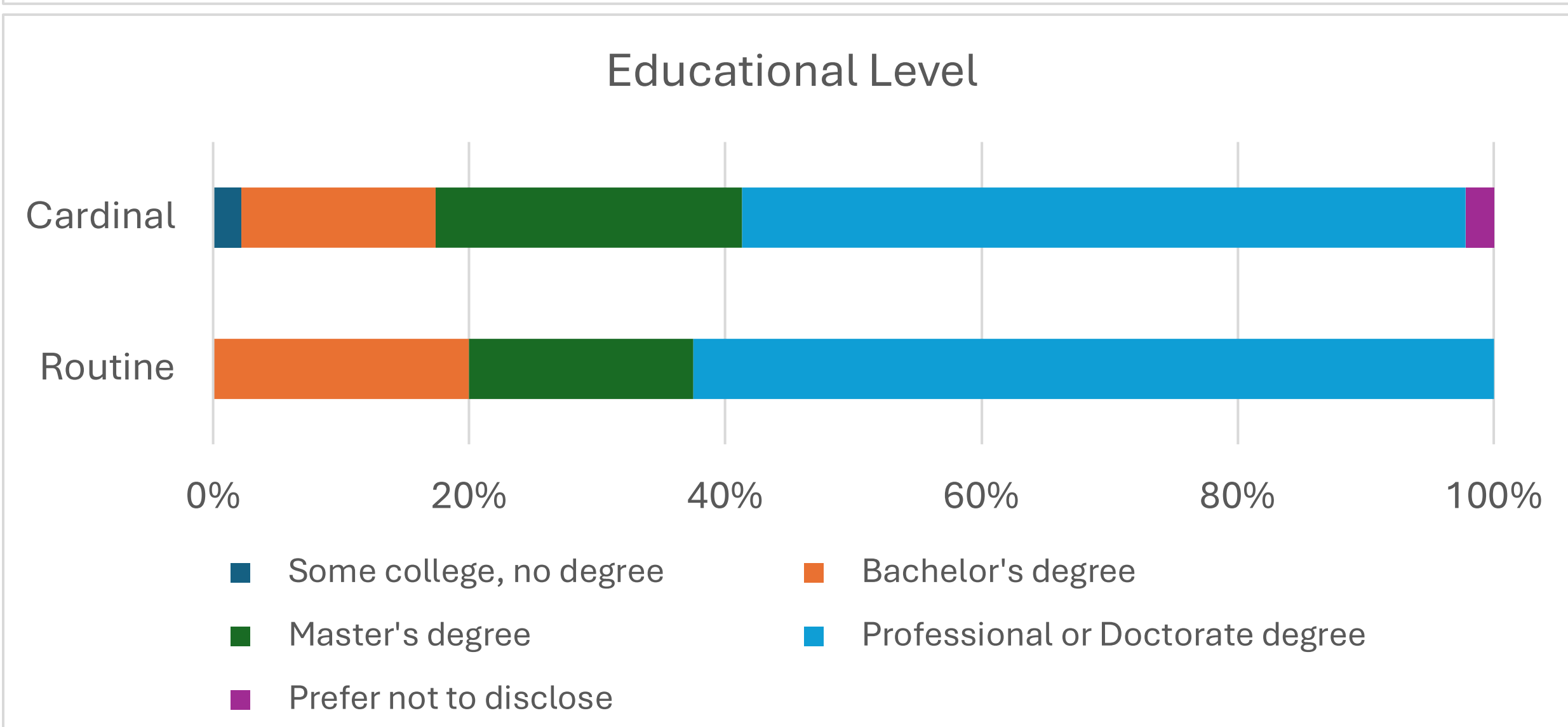
Educational Level
Cardinal
Routine
0%
20%
40%
60%
80%
100%
Some college, no degree
Bachelor's degree
Master's degree
Professional or Doctorate degree
Prefer not to disclose

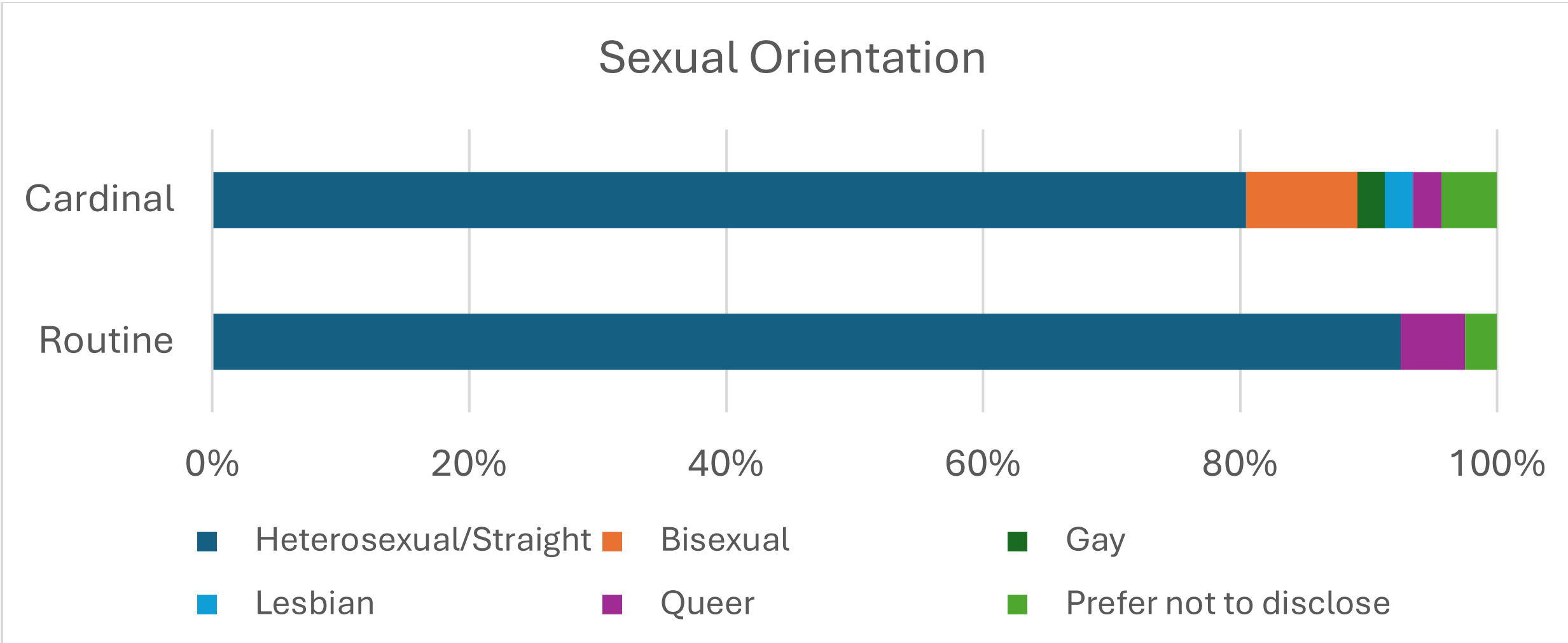
Sexual Orientation
Cardinal
Routine
0%
20%
40%
60%
80%
100%
Heterosexual/Straight
Bisexual
Gay
Lesbian
Queer
Prefer not to disclose

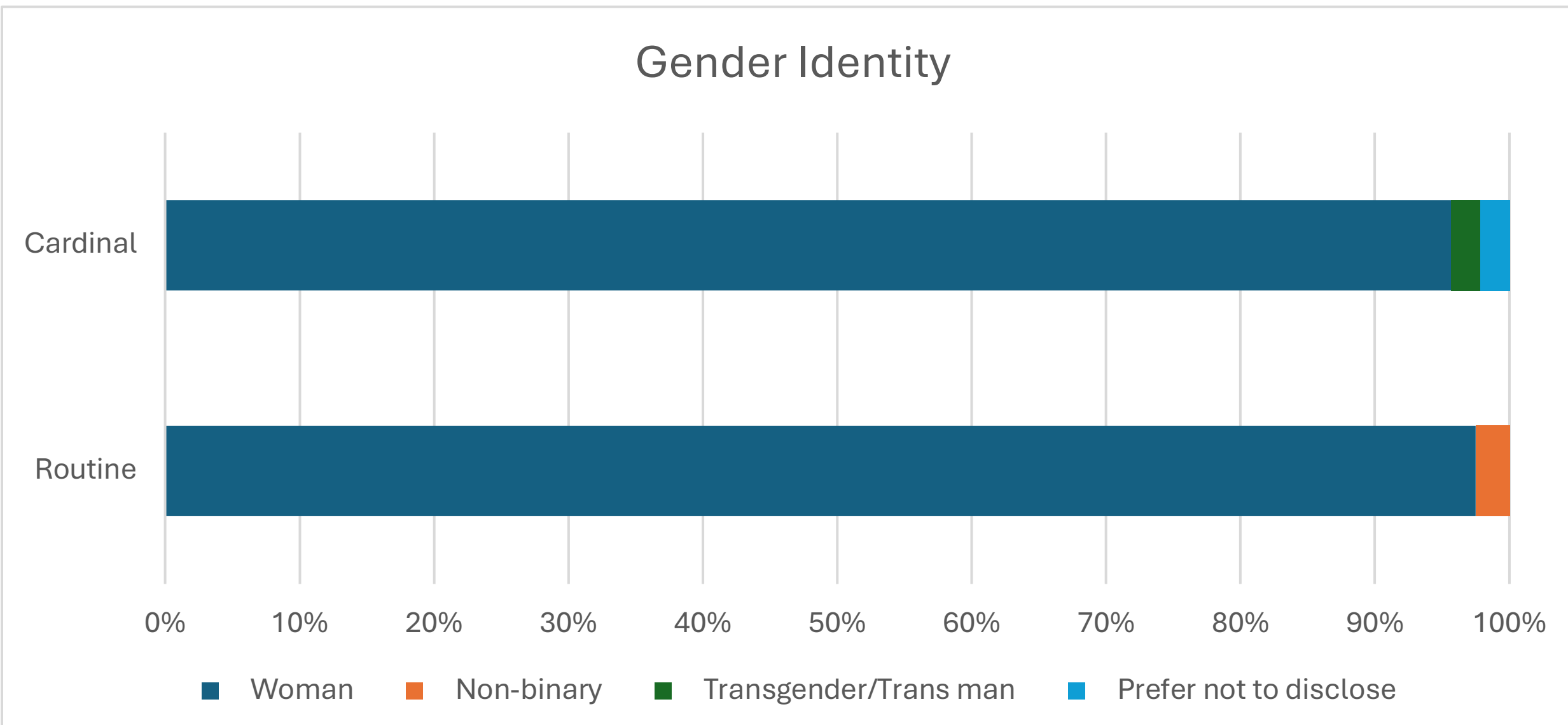
Gender Identity
Cardinal
Routine
0%
10%
20%
30%
40%
50%
60%
70%
80%
90%
100%
Woman
Non-binary
Transgender/Trans man
Prefer not to disclose

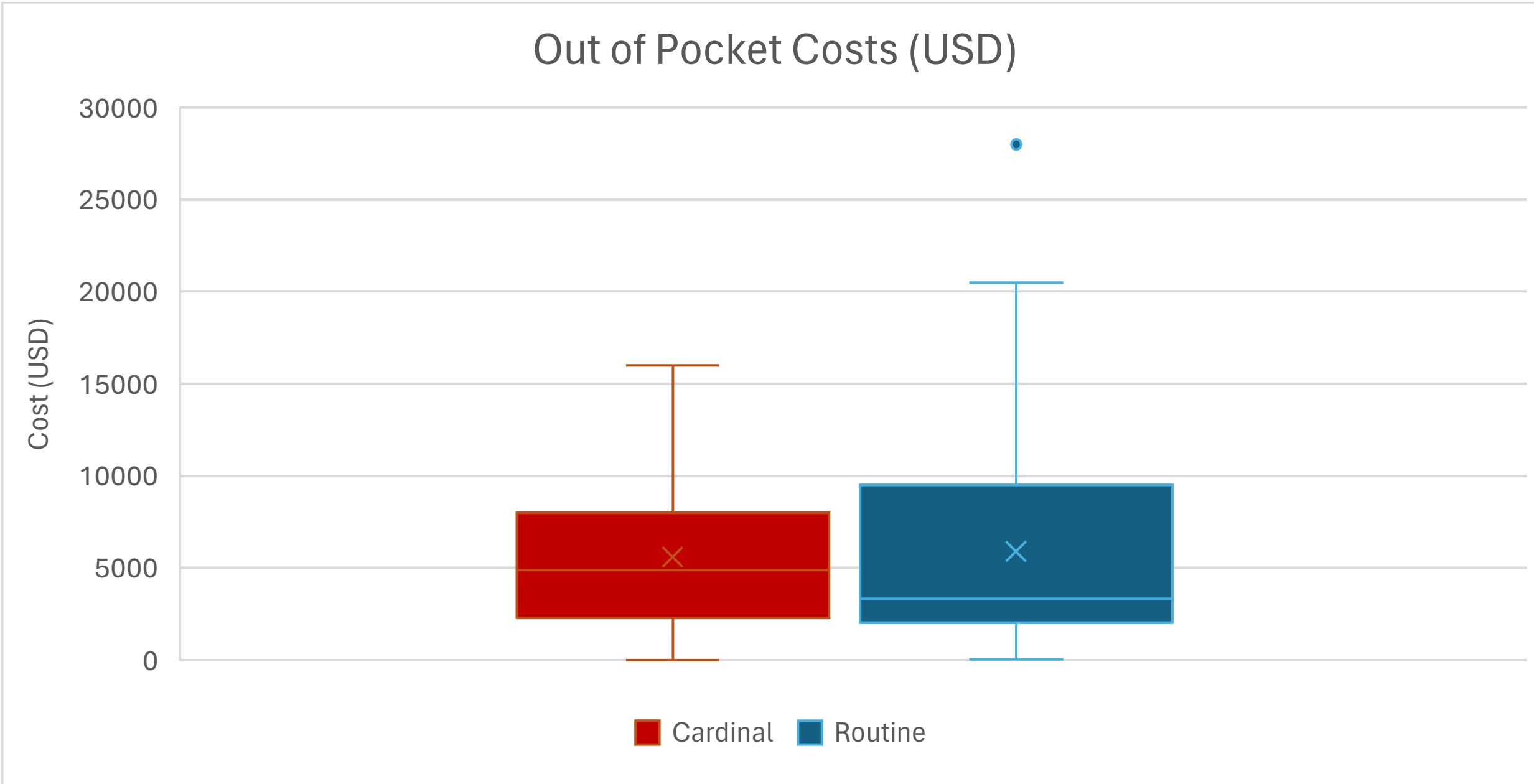
Out of Pocket Costs (USD)
Cost (USD)
30000
25000
20000
15000
10000
5000
0
Cardinal
Routine

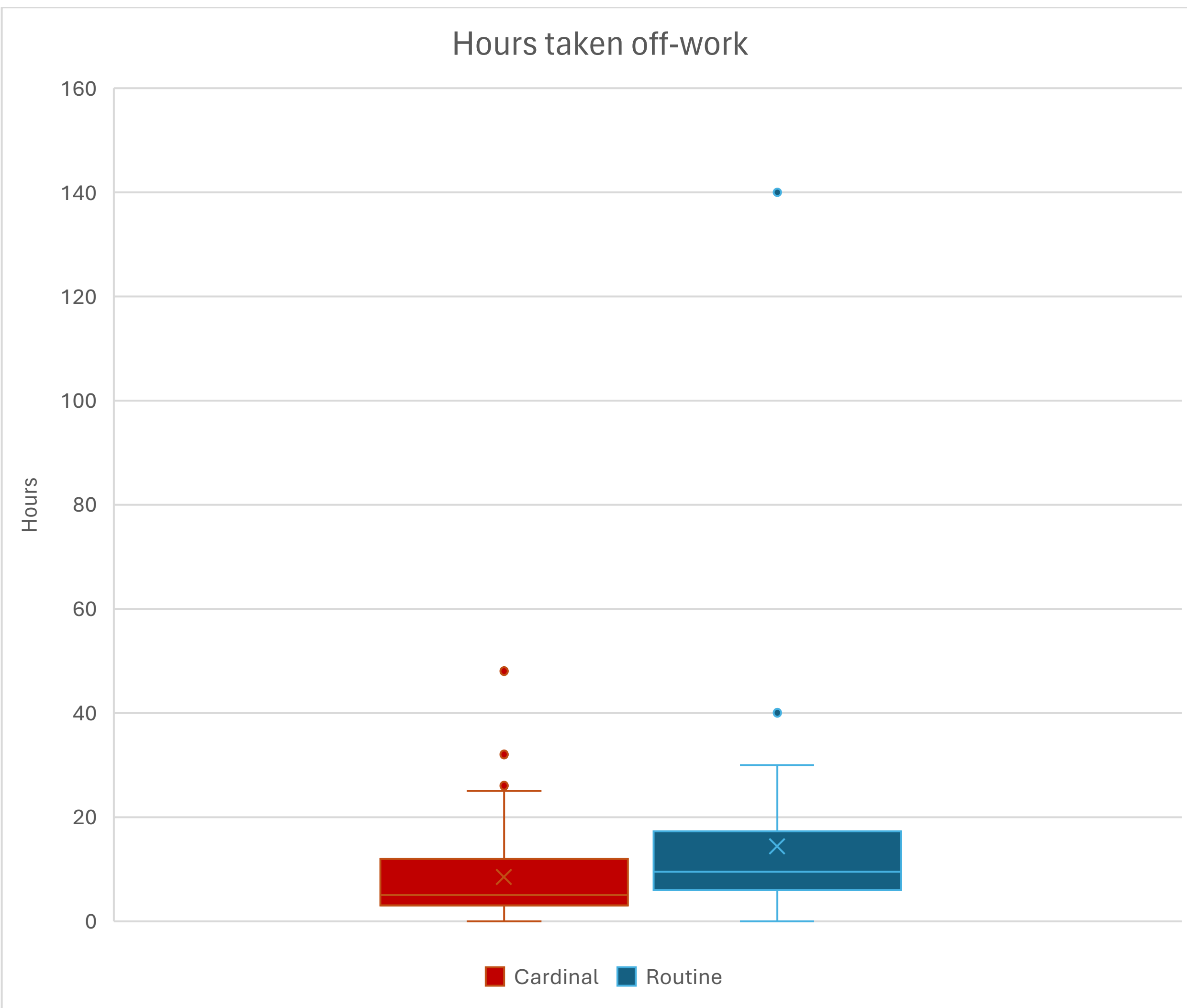
Hours taken off-work
Hours
160
140
120
100
80
60
40
20
0
Cardinal
Routine

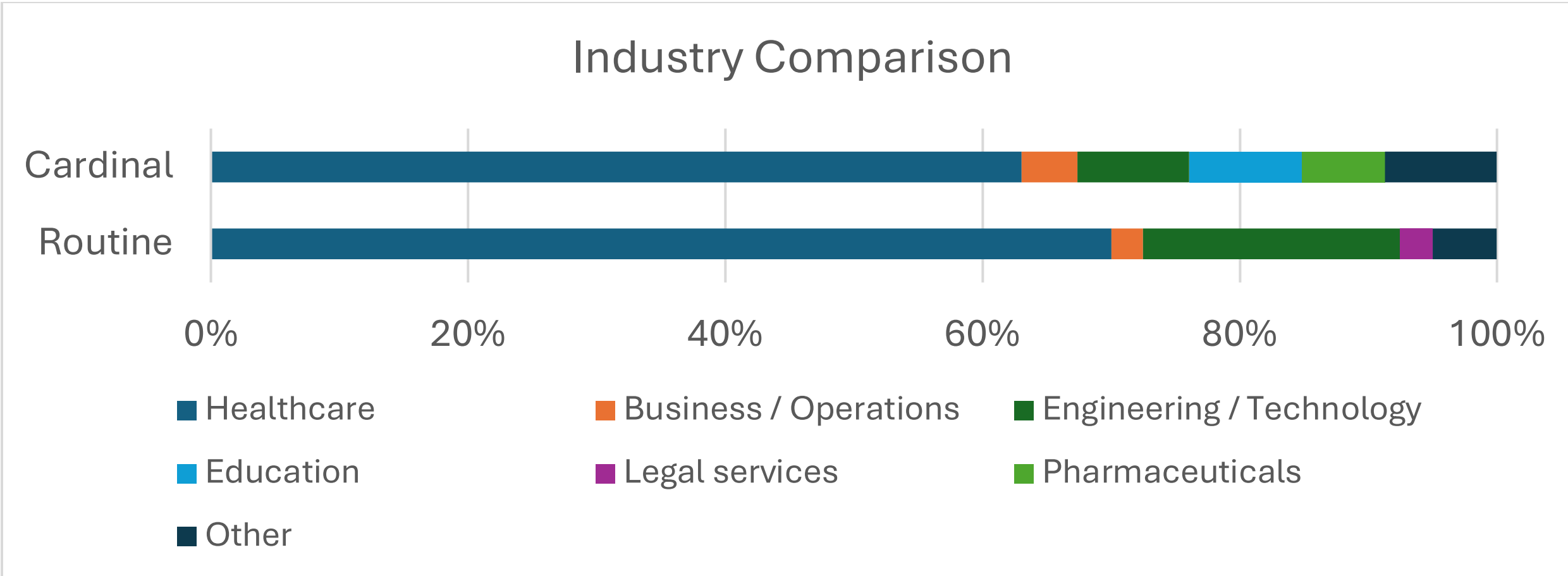
Industry Comparison
Cardinal
Routine
0%
20%
40%
60%
80%
100%
Healthcare
Business / Operations
Engineering / Technology
Education
Legal services
Pharmaceuticals
Other

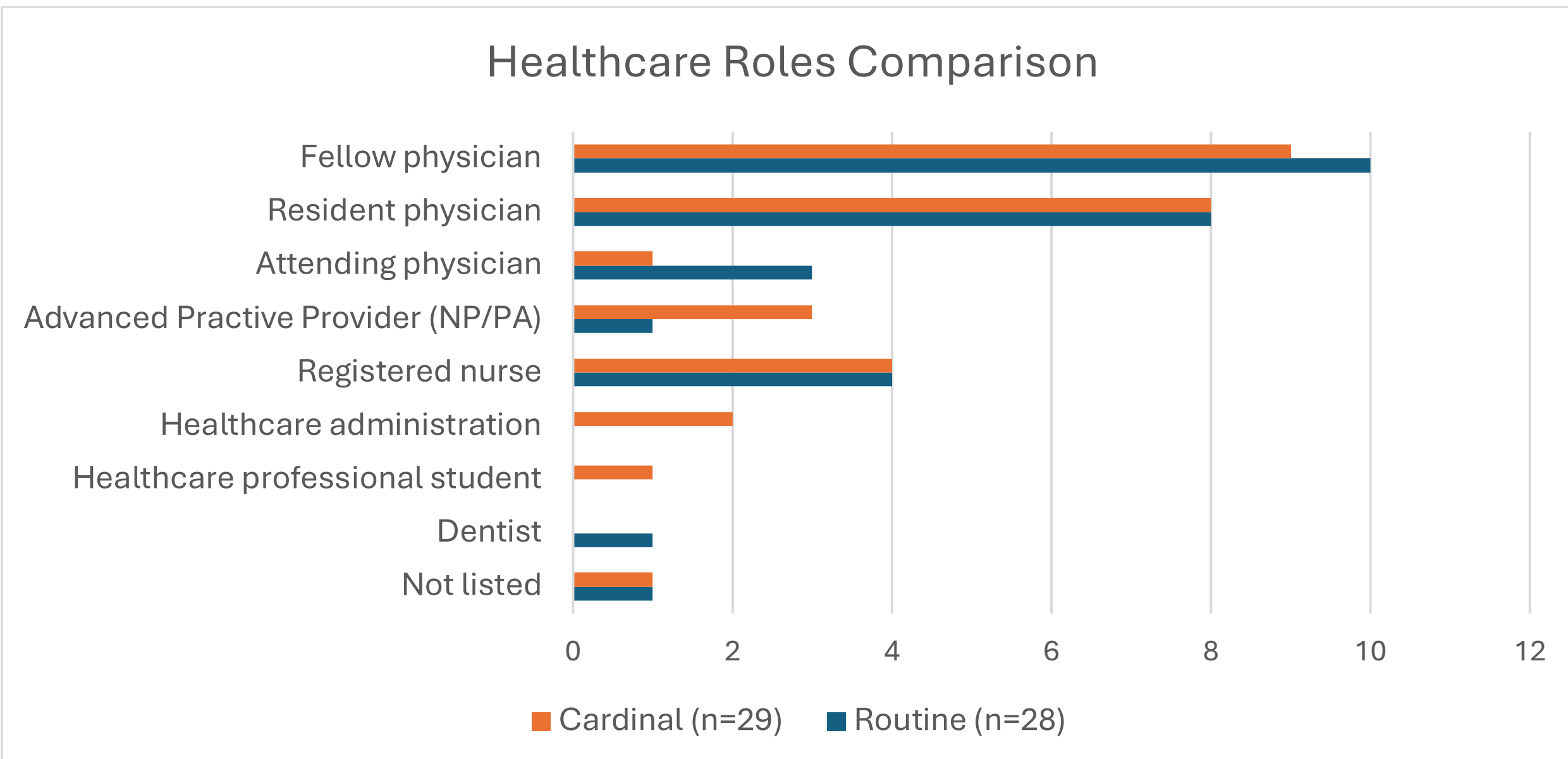
Healthcare Roles Comparison
Fellow physician
Resident physician
Attending physician
Advanced Practive Provider (NP/PA)
Registered nurse
Healthcare administration
Healthcare professional student
Dentist
Not listed
0
2
4
6
8
10
12
Cardinal (n=29)
Routine (n=28)

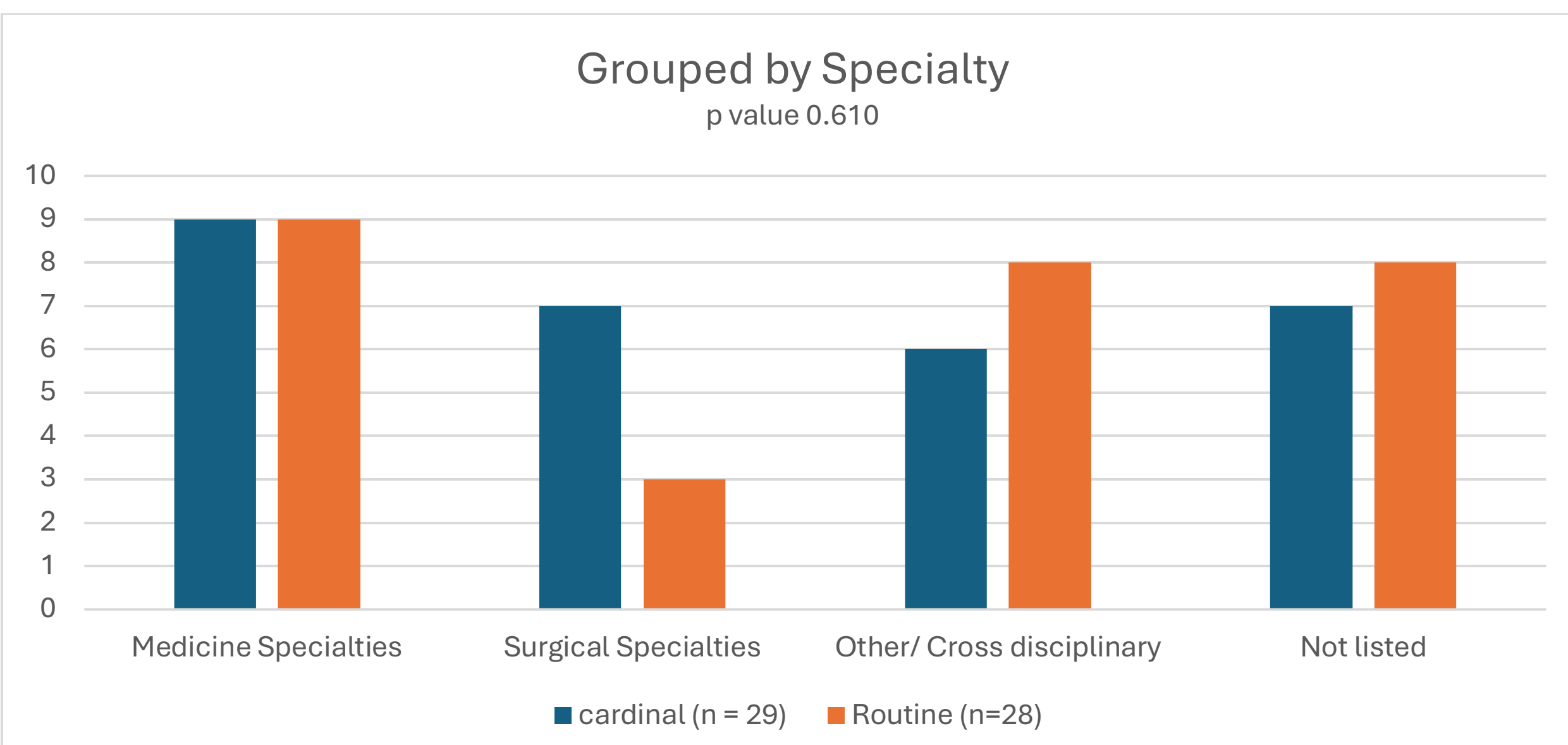
Grouped by Specialty
p value 0.610
10
9
8
7
6
5
4
3
2
1
0
Medicine Specialties
Surgical Specialties
Other/ Cross disciplinary
Not listed
cardinal (n = 29)
Routine (n=28)
