# Supplementary material for: The Cardinal protocol: investigating a simplified, cost-conscious option for oocyte cryopreservation in a prospective noninferiority cohort study
Source: F S Rep. 2026 May 8;7(4):301–12. doi: 10.1016/j.xfre.2026.05.001 (PMC13494622; doi:10.1016/j.xfre.2026.05.001)
Supplement: Supplementary file 3 [file mmc1.docx]

**Supplementary Table 1: Patient Baseline and Cycle Characteristics for 1^st^ Cycles Only (N=118)**

|  | **Cardinal** | | **Routine** | | **p-value** |
| --- | --- | --- | --- | --- | --- |
| **Participants** | 60 | | 58 | | -- |
| Age | 33 (32, 35) | | 32 (31, 34) | | 0.07 |
| AFC | 15 (11, 19) | | 17 (12, 23) | | 0.09 |
| AMH (ng/ml) | 2.56 (1.54, 3.55) | | 3.30 (1.86, 4.68) | | 0.06 |
| Age at retrieval |  | |  | | 0.07 |
| Median (IQR) | 33 (32, 35) | | 32 (31, 34) | |  |
| Min, Max | 21 - 39 | | 27 - 40 | |  |
| Race |  | |  | |  |
| American Indian or Alaska Native | 0 (0%) | | 0 (0%) | | 0.28 |
| Asian | 25 (45.5%) | | 26 (45.6%) | |  |
| Black or African American | 3 (5.5%) | | 4 (7.0%) | |  |
| Native Hawaiian or Other Pacific Islander | 0 (0%) | | 0 (0%) | |  |
| White | 23 (41.8%) | | 19 (33.3%) | |  |
| Other | 2 (3.6%) | | 7 (12.3%) | |  |
| More than one races | 2 (3.6%) | | 1 (1.8%) | |  |
| Unknown | 5 | | 1 | |  |
| Ethnicity |  | |  | | >0.99 |
| Yes | 3 (5.3%) | | 3 (5.3%) | |  |
| No | 54 (94.7%) | | 54 (94.7%) | |  |
| Unknown | 3 | | 1 | |  |
| Distance from home to clinic |  | |  | | 0.65 |
| Median (IQR) | 14 (10, 38) | | 15 (7, 26) | |  |
| Min, Max | 2.4, 2938 | | 2, 64.3 | |  |
| Unknown | 0 | | 2 | |  |
| **Cycles** | **60** | | **58** | | **--** |
| BMI | 23.4 (20.8, 25.3) | | 23.2 (21.5, 25.9) | | 0.49 |
| **Excluding 1 cancelled cycle** | **60** | | **57** | |  |
| Starting FSH dose (IU) | 300 (300, 300) | 350 (300, 450) | | <0.01 | |
| Daily LH dose (IU) | 75 (75, 75) | | 150 (75, 150) | | <0.01 |
| Total FSH dose (IU) | 2400 (2400, 2850) | | 3150 (2500, 4125) | | <0.01 |
| # of US visits | 2 (2,3) | | 5 (5, 6) | | <0.01 |
| # of total labs drawn | 0 (0,1) | | 5 (3, 7) | | <0.01 |
| Trigger Day | 10 (9,11) | | 10 (9,11) | | 0.26 |
| Total Charges (including medications) | $19,630 (19,373, 20,510) | | $24,742 (23,807, 25,729) | | <0.01 |

**Supplementary Table 2: Cardinal vs. Routine Cycles Limited to 1^st^ Cycles Only: Number of Oocytes**

|  | **Cardinal**  **(No. Cycle = 60)** | **Routine**  **(No. Cycle = 57)** | **Crude mean difference (95% CI)** | **Adjusted mean difference* (95% CI)** |
| --- | --- | --- | --- | --- |
| # of oocytes retrieved | 15 (8, 20) | 17 (12, 25) | -2.1 (-5.6, 1.4) | 0.35 (-2.6, 3.3) |
| # of MIIs retrieved | 11 (7, 15) | 11 (7, 17) | -1.2 (-3.9, 1.5) | 0.64 (-1.7, 3.0) |
| # of MIIs and MI-MII | 11 (7, 16) | 12 (8, 19) | -1.3 (-4.2, 1.5) | 0.58 (-1.9, 3.0) |
| # of oocytes frozen | 11 (8, 16) | 12 (8, 19) | -1.1 (-3.9, 1.7) | 0.73 (-1.7, 3.1) |

**Supplementary Table 3: Cardinal vs. Routine Cycles Limited to 1^st^ Cycles Only: Other Secondary Clinical Outcomes**

|  | **Cardinal**  **(No. Cycle = 60)** | **Routine**  **(No. Cycle = 58)** | P value |
| --- | --- | --- | --- |
| Cycle Cancellation | 0 | 1 (1.7%) | 0.49 |
|  | **Cardinal**  **(No. Cycle = 60)** | **Routine**  **(No. Cycle = 57)** | p-value |
| Premature Ovulation | 0 (0%) | 0 (0%) | / |
| OHSS (includes mild) | 2 (3.3%) | 15 (26.0%) | <0.01 |
| Moderate or severe OHSS | 1 (1.7%) | 4 (7%) | 0.52 |
| Ovarian torsion | 0 (0%) | 0 (0%) | / |
| Post-op clinic visit | 1 (1.7%) | 4 (7.0%) | 0.20 |
| Post-op ED visit | 0 (0%) | 2 (3.5%) | 0.24 |
| Surgical complications | 0 (0%) | 0 (0%) | / |

**Supplementary Table 4: Cardinal vs. Routine Cycle Outcomes for Normal Responders (# of patient = 72, # of cycles = 80)**

|  | **Cardinal**  **(No. Cycle = 42)** | **Routine**  **(No. Cycle = 38)** | **Crude mean difference** (95% CI) | **Adjusted mean difference* (95% CI)** |
| --- | --- | --- | --- | --- |
| # of oocytes retrieved | 12 (8, 20) | 13 (9, 20) | -0.32 (-4.0, 3.4) | 0.00 (-3.2, 3.2) |
| # of MIIs retrieved | 10 (6, 14) | 10 (6, 13) | 0.41 (-2.4, 3.2) | 0.88 (-1.6, 3.4) |
| # of MIIs and MI-MII | 10 (7, 14) | 11 (7, 15) | -0.10 (-3.0, 2.8) | 0.27 (-2.3, 2.9) |
| # of oocytes frozen | 10 (7, 15) | 11 (7, 15) | -0.13 (-3.0, 2.7) | 0.32 (-2.2, 2.8) |

*The multivariable model adjusted for patient age, race, ethnicity, BMI, AFC, and AMH.

**Supplementary Table 5: Per-Unit Costs for Services Provided and for Fertility Medications**

| **Service Provided or Medication** | **Per-Unit Cost** |
| --- | --- |
| Transvaginal ultrasounds | $510 |
| Phlebotomy | $57 |
| Bloodwork (E2, LH, P4) | $382 |
| Antagonist (Ganirelix) | $112 |
| Provera (7d prescription) | $5 |
| Clomid (5d prescription) | $23 |
| Gonal-F (450 IU) pen/vial | $420 |
| Gonal-F (300 IU) pen/vial | $280 |
| Menopur (150 IU) | $176 |
| Menopur (75 IU) | $88 |
| Novarel 4000 IU vial | $85 |
| Ovidrel 250mcg | $107 |
| Leuprolide trigger (4mg/0.8mL) | $159 per vial |

**Supplementary Table 6: Post-cycle Survey Outcomes on Patient Satisfaction and Socio-demographic Factors**

|  | Cardinal (46) | Routine (40) | p-value |
| --- | --- | --- | --- |
| On a scale from 1-5, how satisfied were you with your egg-freezing treatment? |  |  | 0.03 |
| 1 | 0 (0%) | 0 (0%) |  |
| 2 | 1 (2.2%) | 6 (15%) |  |
| 3 | 6 (13%) | 8 (20%) |  |
| 4 | 19 (41%) | 18 (45%) |  |
| 5 | 20 (43%) | 8 (20%) |  |
| Satisfaction defined as 4 or 5 |  |  | 0.03 |
| Dissatisfied | 7 (15%) | 14 (35%) |  |
| Satisfied | 39 (85%) | 26 (65%) |  |
| What did you appreciate/like about your egg-freezing experience? Check all that apply. | | |  |
| Outcome or ovarian response (# of eggs) | 25 (54%) | 20 (50%) | 0.69 |
| Cost | 25 (54%) | 5 (13%) | <0.01 |
| Convenience | 33 (72%) | 13 (33%) | <0.01 |
| Staff | 30 (65%) | 30 (75%) | 0.32 |
| Other | 5 (11%) | 3 (7.5%) | 0.72 |
| What did you dislike/feel like could improve your egg-freezing experience? Check all that apply. | | | |
| Outcome or ovarian response (# of eggs) | 17 (37%) | 16 (40%) | 0.77 |
| Cost | 18 (39%) | 25 (63%) | 0.03 |
| Convenience | 6 (13%) | 19 (48%) | <0.01 |
| Staff | 6 (13%) | 6 (15%) | 0.79 |
| Other | 18 (39%) | 12 (30%) | 0.38 |
| Insurance coverage | |  | 0.73 |
| Complete coverage | 14 (30%) | 11 (28%) |  |
| Partial coverage | 24 (52%) | 24 (60%) |  |
| No coverage | 8 (17%) | 5 (13%) |  |
| Total annual household income? |  |  | 0.02 |
| < $100,000 | 13 (28%) | 12 (30%) |  |
| $100,000 - $200,000 | 25 (54%) | 10 (25%) |  |
| $200,000 - $300,000 | 6 (13%) | 9 (23%) |  |
| $300,000 - $400,000 | 0 (0%) | 4 (10%) |  |
| $400,000+ | 1 (2.2%) | 3 (7.5%) |  |
| prefer not to disclose | 1 (2.2%) | 2 (5.0%) |  |
| Income group |  |  | <0.01 |
| Less than $200,000 | 38 (83%) | 22 (55%) |  |
| More than $200,000 | 8 (17%) | 18 (45%) |  |
| Current employment status? |  |  | 0.62 |
| Unemployed | 2 (4.3%) | 1 (2.5%) |  |
| Part-time | 2 (4.3%) | 0 (0%) |  |
| full-time | 41 (89%) | 39 (98%) |  |
| Prefer not to disclose | 1 (2.2%) | 0 (0%) |  |
| Hours taken off-work for appointments, excluding day of retrieval |  |  | 0.03 |
| Median | 5 (3, 12) | 10 (6, 16) |  |
| Mean | 8.5 | 14.3 |  |
| Unknown | 1 | 0 |  |
| How do you identify? |  |  | 0.85 |
| Man | 0 (0%) | 0 (0%) |  |
| Woman | 44 (96%) | 39 (98%) |  |
| Non-binary | 0 (0%) | 1 (2.5%) |  |
| Transgender/Trans woman | 0 (0%) | 0 (0%) |  |
| Transgender/Trans man | 1 (2.2%) | 0 (0%) |  |
| Not listed | 0 (0%) | 0 (0%) |  |
| Prefer not to disclose | 1 (2.2%) | 0 (0%) |  |
| What is your sexual identity? |  |  | 0.23 |
| Asexual | 0 (0%) | 0 (0%) |  |
| Bisexual | 4 (8.7%) | 0 (0%) |  |
| Gay | 1 (2.2%) | 0 (0%) |  |
| Lesbian | 1 (2.2%) | 0 (0%) |  |
| Heterosexual/Straight | 37 (80%) | 37 (93%) |  |
| Queer | 1 (2.2%) | 2 (5.0%) |  |
| Not listed | 0 (0%) | 0 (0%) |  |
| Prefer not to disclose | 2 (4.3%) | 1 (2.5%) |  |
| Heterosexual vs. Not |  |  | 0.11 |
| Heterosexual/Straight | 37 (80%) | 37 (93%) |  |
| Not Heterosexual | 9 (20%) | 3 (7.5%) |  |
